# Numerical Study on the Axial Compressive Behavior of Steel-Tube-Confined Concrete-Filled Steel Tubes

**DOI:** 10.3390/ma17010155

**Published:** 2023-12-27

**Authors:** Xiaozhong Li, Sumei Zhang, Yu Tao, Bing Zhang

**Affiliations:** 1 School of Civil and Environmental Engineering, Harbin Institute of Technology (Shenzhen), Shenzhen 518055, China; lixiaozhong@stu.hit.edu.cn (X.L.); 19b954014@stu.hit.edu.cn (Y.T.); zhangbing2003@hit.edu.cn (B.Z.); 2 Guangdong Provincial Key Laboratory of Intelligent and Resilient Structures for Civil Engineering (Shenzhen), Shenzhen 518055, China

**Keywords:** steel-tube-confinement, concrete-filled steel tubes, stub column, finite element models, confining mechanism, design guidelines

## Abstract

To improve the concrete confinement and mechanical properties of concrete-filled steel tube (CFST) columns, a new configuration of steel-tube-confined concrete-filled steel tube (T-CFST) columns has recently been developed, in which an outer steel tube is employed externally, and the additional tube does not sustain the axial load directly. This preliminary experimental study revealed that, due to the effective concrete confinement by the outer steel tube, the T-CFST column achieves higher compressive strength and more ductile deformation compared to the CFST columns of the same steel ratio. In this study, two finite element (FE) models were developed for the T-CFST cross-section and stub column, respectively. The numerical study results revealed that the concrete can be constrained by the outer steel tube at the beginning of loading and the outer steel tube hoop stress can reach its yield strength at the column’s compressive strength, showing its effective confinement to the concrete. Numerous data were generated by the developed FE model to cover a wide range of parameters. Based on that, the calculation methods for the stress components of the inner and outer steel tubes are proposed. Finally, a suitable prediction method is proposed, utilizing the superposition method to determine the compressive strength of the T-CFST stub column, and the results of the calculation method and FE model agree well with each other. This research is the basis for promoting further research of T-CFST columns.

## 1. Introduction

Concrete-filled steel tube (CFST) columns have been widely used in engineering practice for their excellent mechanical properties resulting from the composite action between the steel tube and the infilled concrete [1,2,3]. However, the confinement of concrete by the steel tube is not achieved and developed until the steel tube enters its elastoplastic stage. Meanwhile, the steel tube directly sustains the axial load, and the longitudinal stress is fully developed, resulting in reduced hoop stress in the steel tube and insufficient confinement to the concrete [4]. Due to the delayed and inadequate concrete confinement by the steel tube, CFST columns tend to suffer from poor load-bearing capacity and shear failure.

To improve the magnitude and efficiency of concrete confinement by the steel tube, many different types of CFST columns have been developed by changing their configuration. The steel tube in the steel-tube-confined concrete (T-C) columns is prevented from directly sustaining the axial load by cutting it off at the column ends, leading to enhanced confinement of the concrete core [5,6,7]. Moreover, the infilled plain concrete can be replaced by reinforced concrete (RC) [8,9,10] or steel-reinforced concrete (SRC) [11,12]. However, shear failure can still be observed in some cases for the CFST and T-C columns, even with RC or SRC infilling [13]. Besides, external confinement is employed in the form of steel tubes [14,15,16,17,18], fiber reinforced plastic (FRP) tubes [19,20], FRP jackets [21,22,23,24], FRP rings [25,26,27,28], or discrete stirrup [29,30,31,32,33,34,35,36], which enhances the mechanical properties of the CFST columns to a certain extent.

**Figure 1:**
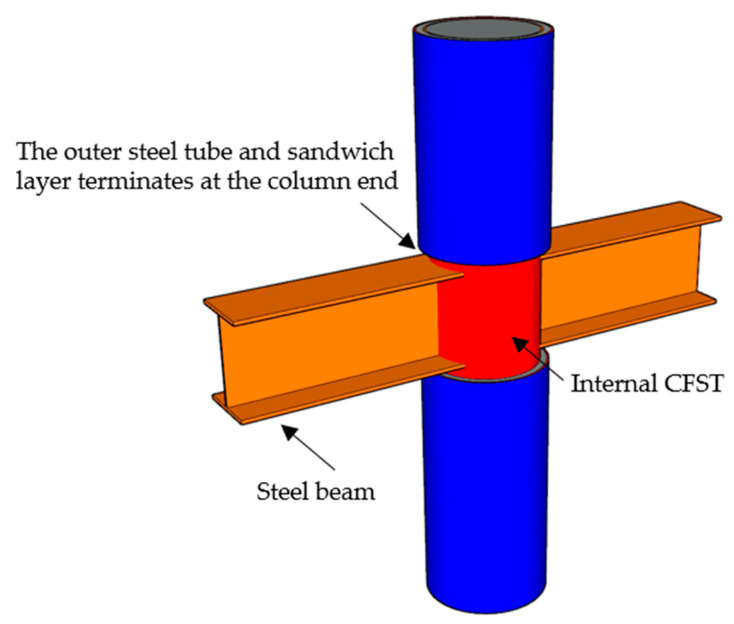
Typical T-CFST column.

Apart from that, the newly developed steel-tube-confined CFST (T-CFST) columns have been proven to be effective in improving load-bearing capacity and preventing CFST columns from experiencing shear failure [13,37,38]. The T-CFST columns are composed of the internal CFST, an outer steel tube, and a sandwich layer (Figure 1). Both the outer steel tube and sandwich layer terminate at the column ends and do not enter the beam–column joint, which differentiates the T-CFST from the concrete-filled double-skin steel tube (CFDST) columns. Therefore, the outer steel tube is prevented from directly sustaining the axial load, leading to the maximized confinement to the concrete core. Meanwhile, the beam–column joint is kept the same as the joints of the beam and CFST columns, which are simple to construct. In addition, the new configuration is also able to retrofit and strengthen deficient CFST columns. Considering the protection provided by the external jacketing, the resistance to impact, blast, and seismic loads as well as to fire and corrosion can be improved further for internal CFST columns. This preliminary experimental study shows that, compared to the CFST and T-C columns with the same steel ratio employed, the T-CFST column achieves 20.6~42.4% higher axial compressive strength and that shear failure is prevented [13].

In recent years, experimental studies have been conducted to determine the mechanical properties of T-CFST columns [13,37,38]. However, further research should be conducted to illuminate the working mechanism of each component. Furthermore, the influence of the critical parameters also should be illustrated and discussed within a broader scope. In addition, a suitable prediction method also needs to be developed, and design suggestions also must be provided. In response to that, a rigorous numerical study will be conducted in this paper for T-CFST columns under axial compression. A suitable constitutive model is proposed for the concrete material considering the confinement by the outer and inner steel tubes. Then, based on the developed finite element (FE) model, the confining mechanism will be determined, numerous data will be generated to cover a wide range of parameters, and the configuration of the T-CFST columns will be investigated. After that, design suggestions will be given to specify the column configuration and a corresponding calculation method will be developed for the T-CFST columns.

## 2. FE Modelling

### 2.1. General Description

To maximize the concrete confinement by the steel tubes in the T-CFST column, the external jacketing is terminated at the column ends to avoid directly sustaining the axial load.

**Figure 2:**
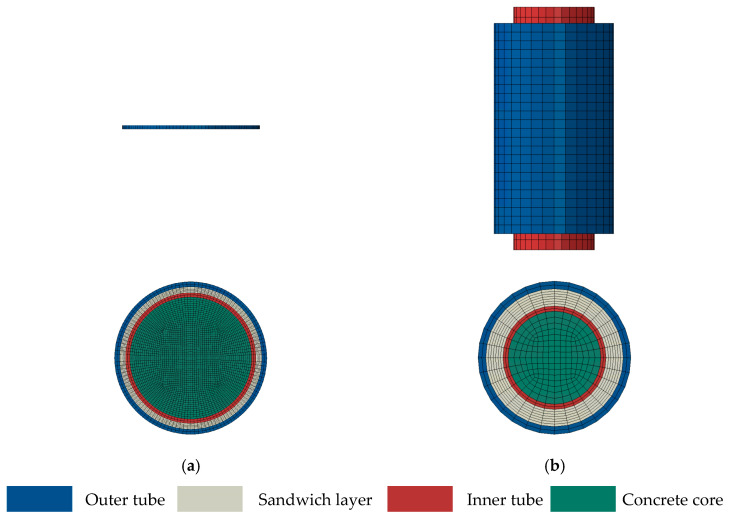
FE models of the T-CFST column: (**a**) cross-section; (**b**) stub column.

However, due to the friction over the external jacketing and inner tube interface, a partial axial load is transferred to the external jacketing. A continuously increased axial load is obtained for the external jacketing as the interfacial height increases. The changed axial load leads to unevenly distributed longitudinal and horizontal outer tube stresses along the column height, further affecting the development of stress components and mechanical performance. To eliminate the influence of friction on the confinement mechanism, an FE model of the cross-sectional T-CFST column was established at first, and the mutual influence of the tubes and concrete was revealed. After that, an FE model of the T-CFST stub column was developed to determine the mechanical properties of the axially loaded T-CFST column. The cross-sectional and stub column’s FE models are shown in Figure 2, and the height of the former model is taken as 1/300 of the inner tube diameter while the height of the stub column is three times the inner tube diameter.

### 2.2. Material Models

#### 2.2.1. Steel Material

The typical five-stage stress (*σ*)–strain(*ε*) model [39] is employed for the tubes. The key parameters, including the yield strength *f*_y_, ultimate strength *f*_u_, elastic modulus *E*_s_, and Poisson’s ratio *μ*_s_, are given as the measured values to develop the FE model for the experimental specimens. For the FE models in the parameter study, the values of 206 GPa and 0.2 are taken for the *E*_s_ and *μ*_s_, respectively.

#### 2.2.2. Concrete and Sandwich Material

Due to the sandwich material being restrained by the outer tube only, the three-stage stress(*σ*)–strain(*ε*) model is employed, which is proposed by Tao [40] to simulate the core concrete in CFST columns. Compared to unconfined concrete, the constitutive model is determined based on two key parameters, which are related to the concrete confinement. The first parameter is the confinement index *ξ*, while the second one is the concrete confining stress at the endpoint of the plateau stage *f*_B_. The sandwich material in the T-CFST columns is confined by the outer tube only, and thus the two parameters can be determined as *ξ* = *f*_y2_*A*_s2_/*f*_c2_$A_{c2}^{\prime }$ and Equation (1), respectively, in which *f*_y2_ is the outer tube strength while *f*_c2_ is the sandwich material strength. *A*_s2_, and $A_{c2}^{\prime }$ are the outer tube area and the area surrounded by the outer tube. Comparatively, the confinement index of the core concrete is determined as *ξ* = (*f*_y2_*A*_s2_ + *f*_y1_*A*_s1_)/*f*_c1_*A*_c1_, in which *f*_y1_ is the inner tube strength, *f*_c1,_ and *A*_c1_ is the core concrete strength and area. The experimental results showed that the outer tube hoop stress can reach its yield strength at the column’s strength, and the concrete confining stress *f*_B_ thus can be determined by Equation (2), where *D*_2_ and *t*_2_ are the outer tube diameter and thickness, while *D*_1_ and *t*_1_ are the corresponding values for the inner tube.

(1) $$f_{B}=\frac{(1+0.027f_{y2})e^{-0.02D_{2}/t_{2}}}{1+1.6e^{-10}{\left(f_{c2}\right)}^{4.8}}$$

(2) $$f_{B}=\frac{(1+0.027f_{y2})e^{-0.02D_{1}/t_{1}}}{1+1.6e^{-10}{\left(f_{c1}\right)}^{4.8}}+\frac{2t_{2}f_{y2}}{D_{1}-2t_{1}}$$

### 2.3. Elements and Meshing

Four-node shell elements with reduced integration (S4R) were employed for the tubes and FRP wrapping, while eight-node solid elements with reduced integration (C3D8R) were adopted for the concrete core and sandwich layer material. To balance the simulation time and analysis accuracy of the FE model, mesh sensitivity evaluation work was conducted carefully. According to the results, the radial size of the element is taken as 1/16 of the inner tube diameter while the longitudinal size is twice that.

### 2.4. Interfacial Properties and Boundaries

Based on the experimental observation, a relative sliding can be seen between the external jacketing and internal CFST for the axially loaded T-CFST stub column. A surface-to-surface contact model with friction was employed to simulate the interfacial behavior over the concrete–steel interfaces. “Hard contact” and “Coulomb friction modeling” are adopted in the normal and tangential direction, respectively. According to the existing experimental results [41,42,43], the friction factor is within 0.2~0.7 for the concrete–steel interface, and a value of 0.6 is taken in this paper.

Considering the symmetry of the stub column in the axial direction, a 1/2 FE model was developed for the cross-sectional FE model, in which the axial and rotational displacement at the bottom surface were restrained while the vertical displacement was applied at the top surface. The FE model for the stub column was also established. Two reference points were placed and coupled with the bottom and top surfaces, for which all the freedoms were restrained except for the longitudinal freedom of the top surface.

## 3. Verification

The experimental results in reference [37,38] are taken to determine the accuracy of the FE model, including the load–longitudinal strain curve and local strain/stress development.

### 3.1. Load–Longitudinal Strain Curve

**Figure 3:**
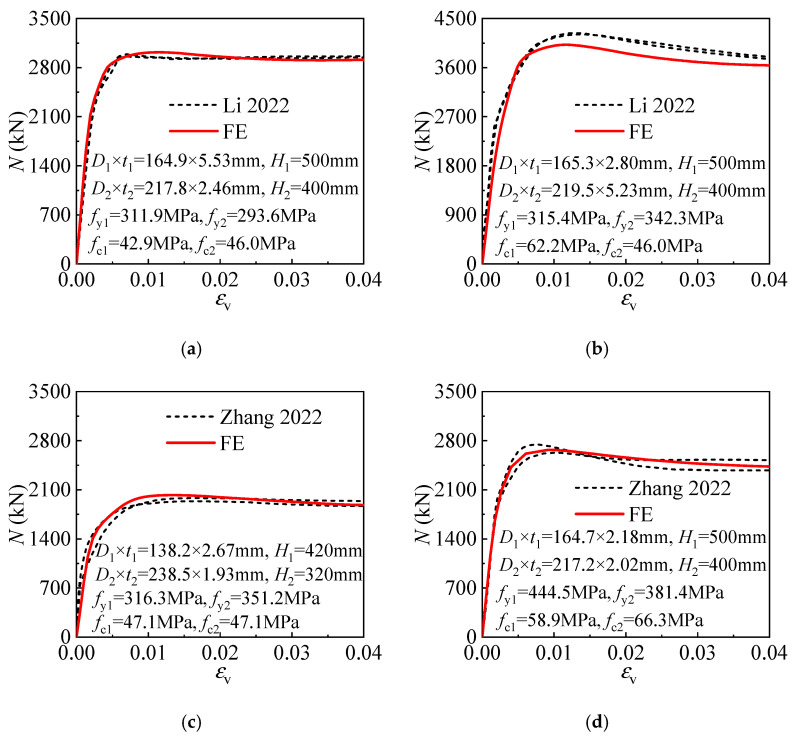
Comparison of the load(*N*)–longitudinal strain(*ε*_v_) curves of the test [37,38] and FE model: (**a**) TF-5.5-2.5; (**b**) TF#-2.75-5.5; (**c**) T-II-2.5-1.5-50; (**d**) T-I-2.5-2.0-50.

The comparison of the experimental and calculated load(*N*)–longitudinal strain(*ε*_v_) curves is given in Figure 3, in which *H*_1_ and *H*_2_ are the inner and outer tube height. The load–longitudinal strain curves obtained by the test and FE model fit well with each other, showing the FE model is satisfactory in predicting the compressive behavior of the T-CFST stub column.

**Figure 4:**
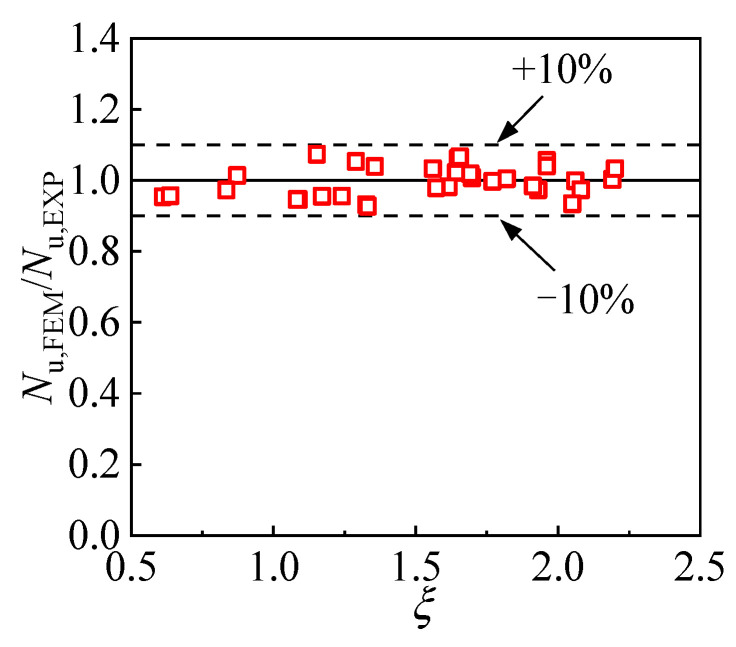
Comparison of compressive strength of the test and the FE model.

The compressive strength obtained by the test and FE model for specimens in a larger parameter scope was given and compared in Figure 4, in which *N*_u, FEM,_ and *N*_u, EXP_ are the results of the test and FE analysis. In total, the data for 33 T-CFST stub columns were included in the comparison, and the confinement index was within 0.61~2.20, covering the usual range in engineering practice. The error of the calculated compressive strength was less than 10%, with the average ratio being 0.997 and the coefficient of variation (COV) being 0.041. The comparison reveals that the established FE model is suitable for determining the T-CFST stub column’s compressive strength.

### 3.2. Strain and Stress Development

**Figure 5:**
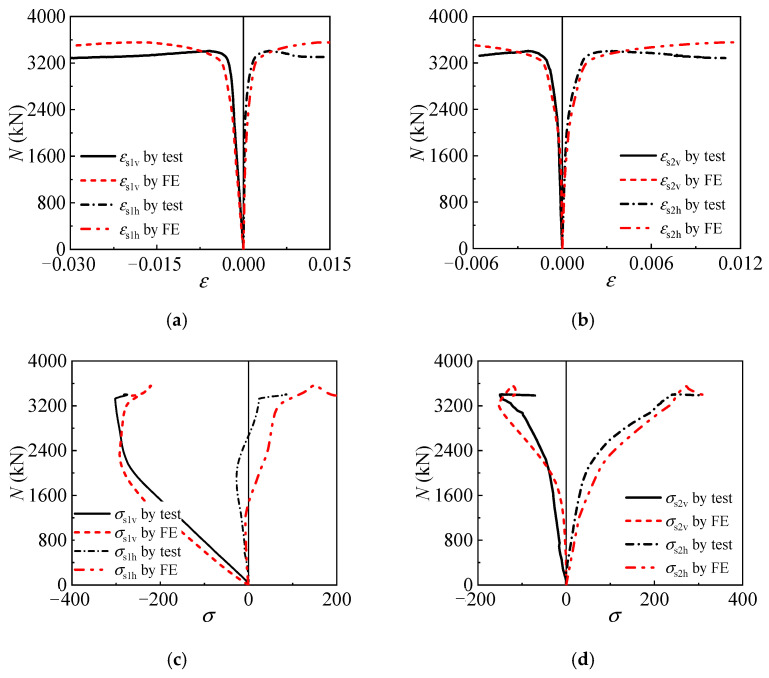
Validation of the FE model: (**a**) inner tube strain; (**b**) outer tube strain; (**c**) inner tube stress; (**d**) outer tube stress.

The strain and stress component of the tubes in the T-CFST stub column is the basis for illustrating the confinement mechanism, and the tested and calculated results are compared in Figure 5. *ε*_s1v_ and *ε*_s1h_ are the mid-height cross-sectional inner tube longitudinal and horizontal strain, respectively, while *ε*_s2v_ and *ε*_s2h_ are the corresponding values of the outer tube. *σ*_s1v_ and *σ*_s2v_ are the inner and outer tube longitudinal stress, while *σ*_s1h_ and *σ*_s2h_ are the horizontal stress. Both the strain and stress development of the tubes can be predicted precisely by the FE model, and all the tested and calculated curves agree well with each other. The comparison again validates the accuracy of the FE model in illustrating the mutual influence of different components and the working mechanism of the axially loaded T-CFST columns.

## 4. Confinement Mechanism

Deep insight into the working mechanism is beneficial for clarifying the compressive behavior of the T-CFST columns, and a cross-sectional FE model with typical parameters was employed to eliminate the influence of the interfacial friction; after that, the influence of friction was illustrated by a T-CFST stub column’s FE model with the same parameters.

### 4.1. Cross-Sectional FE Model

To compare the axial compressive performance and working mechanism between the T-CFST column and the ordinary CFST column, three cross-sectional FE models were developed for the T-CFST and CFST columns. The key parameters of these FE models are shown in Table 1.

#### 4.1.1. Overall Deformation

**Figure 6:**
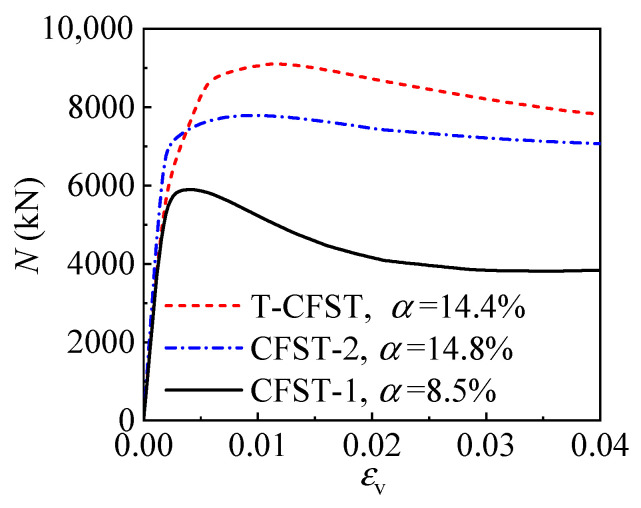
The load(*N*)–longitudinal strain(*ε*_v_) curves of the typical columns.

With a lower steel ratio (*α* = 8.5%) employed, inadequate concrete confinement is obtained for CFST-1. The compressive strength is only 5900.4 kN, and then the axial load drops rapidly, indicating the poor deformation ability of CFST-1 (Figure 6). By increasing the steel ratio to 14.8%, enhanced concrete confinement is achieved for the CFST-2, leading to a 32.0% higher strength. Comparatively, the T-CFST column with the steel ratio of 14.4% achieves a significantly improved compressive strength of 9055.2 kN, which is 53.5% and 16.2% higher than CFST-1 and CFST-2, respectively. Meanwhile, compared to the CFST-1 column, both the CFST-2 and T-CFST column achieve better ductility.

**Figure 7:**
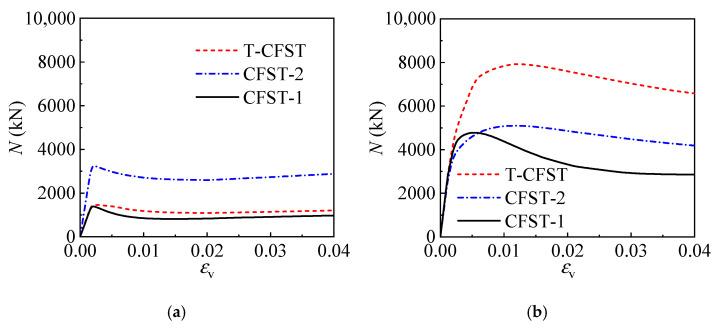
Comparison of the load(*N*)–longitudinal strain(*ε*_v_) curves of the inner tube and concrete core in the typical columns: (**a**) inner tube; (**b**) concrete.

To clarify in depth the reasons for the differences in the compressive behavior of the different column types, Figure 7 depicts the development of the longitudinal deformation as the axial load increases for the steel and concrete components of the three columns. Compared to CFST-1, a thicker tube is employed for CFST-2, and the load sustained by the tube is increased by 128.2%. T-CFST is obtained by setting an outer tube outside the internal CFST portion, and the additional tube cannot sustain the axial load. Due to the confinement by the additional tube, the load sustained by the inner tube decreases at a much slower rate after the peak load. Meanwhile, due to the sufficient concrete confinement by the outer tube, significantly enhanced mechanical properties are achieved for the core concrete in T-CFST (Figure 7a). Compared to CFST-1, the axial load carried by the concrete core of T-CFST at the peak load achieves 7833.3 kN with a significant improvement of 66.5%. However, the effect of increasing the steel content of the inner tube is limited in improving the concrete strength. When the load reaches the column’s compressive strength, compared with CFST-1, the load borne by concrete can only be increased by 7.6% for CFST-2. The above comparison shows that the increasing steel ratio has a margin effect on concrete confinement for CFST columns, and the compressive behavior is thus improved slightly; meanwhile, the concrete is effectively confined by the outer tube, leading to the significantly enhanced mechanical properties of the core concrete, and the T-CFST column achieves better axial compressive behavior.

#### 4.1.2. Confining Process

**Figure 8:**
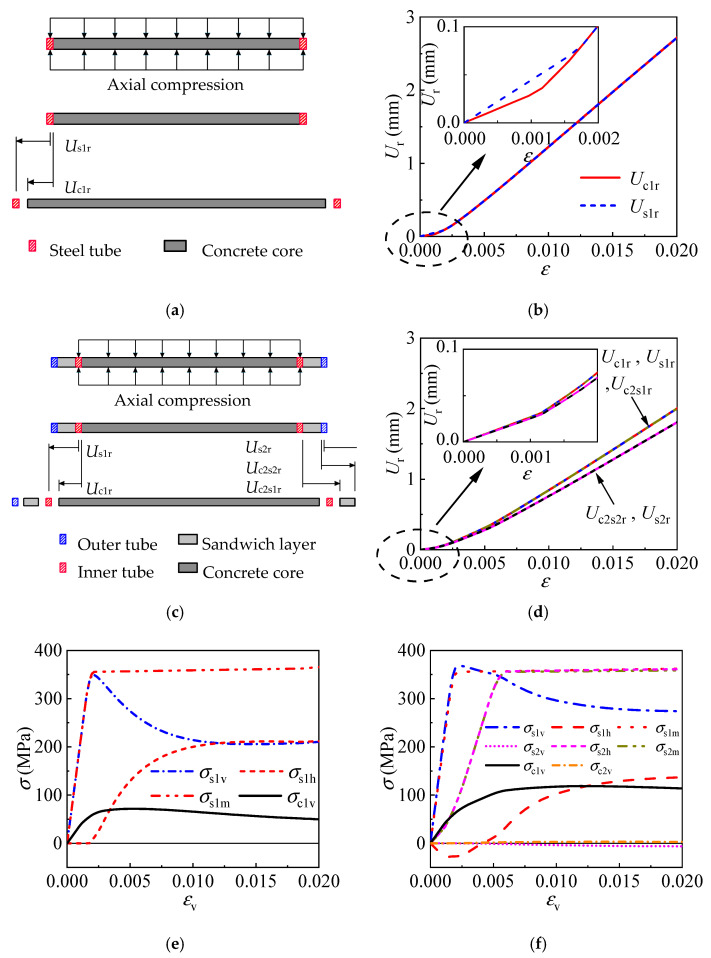
Confining mechanism of the typical columns: (**a**) loading diagram of the CFST-1 column; (**b**) radial deformation of the CFST-1 column; (**c**) loading diagram of the T-CFST column; (**d**) radial deformation of the T-CFST column; (**e**) stress development of the CFST-1 column; (**f**) stress development of the T-CFST column.

The internal CFST is restrained effectively by the outer tube for the T-CFST, leading to significantly enhanced compressive strength and ductility. To better understand the confinement by the outer tube, the difference in the confinement mechanism of the T-CFST and ordinary CFST column will be further compared and discussed in depth. The radial deformation and stress development of the two kinds of columns are shown in Figure 8, in which *U*_r_ is the radial deformation; *U*_c1r_, *U*_s1r_, and *U*_s2r_ are the radial deformation of the concrete, inner tube, and outer tube, respectively; *U*_c2s1r_ and *U*_c2s2r_ represent the radial deformation of the sandwich material adjacent to the inner and outer tube. At the very beginning of loading for CFST-1, a larger Poisson’s ratio of 0.3 is achieved for the steel material compared to the concrete material (0.2), and the tube undergoes greater radial deformation than the concrete core and they are separated as shown in Figure 8b. At this stage, no concrete confinement is provided by the tube (Figure 8e). As the loading continues, the concrete core begins to develop plasticity and the radial deformation increases rapidly; the tube and concrete expand consistently as the axial strain reaches 0.018; after that, the two portions begin to deform cooperatively, and concrete is confined by the tube, and the tube achieves equivalent stress of 0.947*f*_y1_; with further increase in the axial strain, the tube hoop stress keeps increasing while its longitudinal stress decreases rapidly as shown in Figure 8e. However, no axial load is directly sustained by the outer tube for T-CFST, hence producing almost no radial deformation, while the internal CFST bears the axial load and produces radial deformation, pushing the external jacketing to develop radial deformation cooperatively. Therefore, the concrete confinement is derived and developed (Figure 8d); after that, the outer tube hoop stress increases rapidly until the outer tube yields (Figure 8f); then, the outer tube hoop stress remains unchanged, whereas the inner tube hoop stress begins to increase rapidly, providing continuously improving concrete confinement. Moreover, the sandwich material sustains the confining stress by the outer tube horizontally, leading to radial compressive deformation. The radial deformation difference between the sandwich material adjacent to the inner and outer tube also increases gradually as the confinement by the outer tube improves (Figure 8d). The above comparison reveals that the concrete is confined by the tube only after developing plasticity for the CFST, and the confinement is quite small. However, for the T-CFST column, concrete confinement by the outer tube is developed at the very beginning of loading; at the same time, the outer tube can be yielded horizontally, leading to greatly enhanced concrete confinement and significantly improved mechanical properties of the T-CFST column.

#### 4.1.3. Stress Development

**Figure 9:**
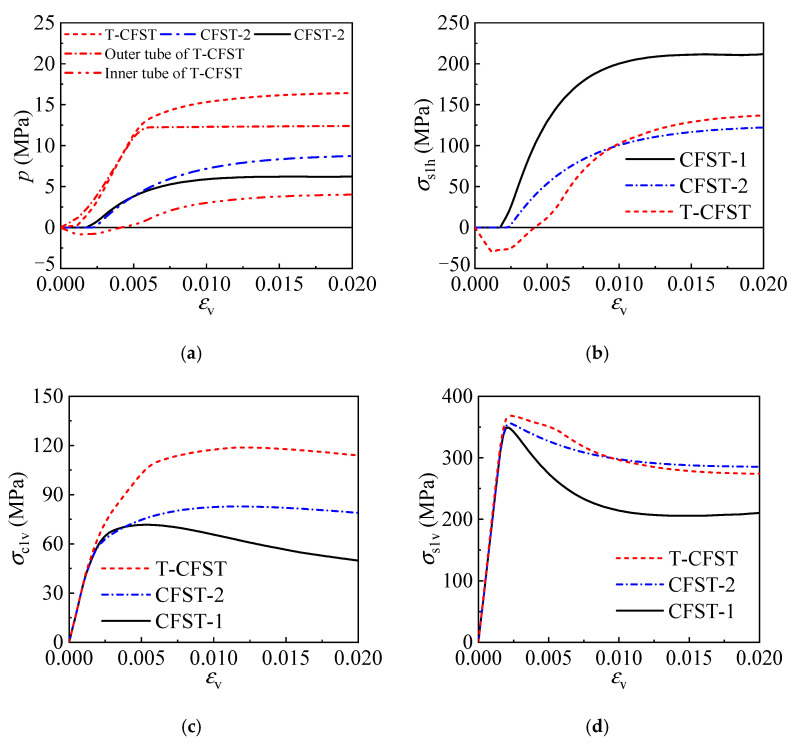
Stress development of the different portions: (**a**) concrete confining stress; (**b**) inner tube hoop stress; (**c**) concrete longitudinal stress; (**d**) inner tube longitudinal stress.

The stress development for the concrete and inner tube is depicted in Figure 9, further illustrating the working mechanism of the T-CFST column. Meanwhile, the stress development of the CFST column is also given in Figure 9 for comparison. The former analysis indicates that the confinement is provided by the outer tube from initial loading (Figure 9a); as the axial strain increases, the concrete confinement by the outer tube enhances rapidly until the tube yields; thereafter, a continuously enhanced confinement is achieved by the inner tube instead, significantly improving the concrete properties. However, during the initial loading stage of ordinary CFST columns, the inner tube hoop stress is zero, indicating no confinement is offered to the concrete core (Figure 9b); after the tube develops plastic deformation, the concrete begins to be restrained by the tube. Meanwhile, the tube achieves a fully developed longitudinal stress and a slowly increased hoop stress, which cannot effectively constrain the concrete core and has a slight influence on the mechanical properties of the CFST column. Figure 9c shows the longitudinal stress of the core concrete for different columns. Significantly improved strength and ductility are obtained for the concrete of the T-CFST owing to the dual confinement by the inner and outer tubes. Compared to CFST-1 and CFST-2, the concrete strength is increased by 65.7% and 43.4% for T-CFST, respectively. Lateral confinement is also provided to the inner tube by the outer tube of the T-CFST column, leading to a horizontally compressed inner tube during the initial loading stage. The inner tube hoop stress changes from compression to tension only after the inner tube yields, and the stress is significantly lower than that of the ordinary CFST columns (Figure 9b) before the column’s compressive strength. The reduced hoop stress leads to a much more fully developed longitudinal stress. Therefore, the inner tube longitudinal stress reaches 1.04 times its yield strength (Figure 9d). Additionally, the inner tube longitudinal stress decreases at a much slower rate than that of the CFST column, which further improves the column’s ductility.

### 4.2. Stub Column FE Model

The analysis result of the cross-sectional FE model reveals that the outer tube in the T-CFST column does not directly bear an axial compressive load but can effectively confine the internal CFST, significantly improving the column’s compressive strength and deformation abilities. However, the external jacketing still bears a partial load as the load can be transferred over the interface between the sandwich layer and the inner tube, affecting the stress development of the external jacketing, especially the outer tube. Therefore, a T-CFST stub column FE model is established to determine the effect of friction on the development of the outer tube stresses.

**Figure 10:**
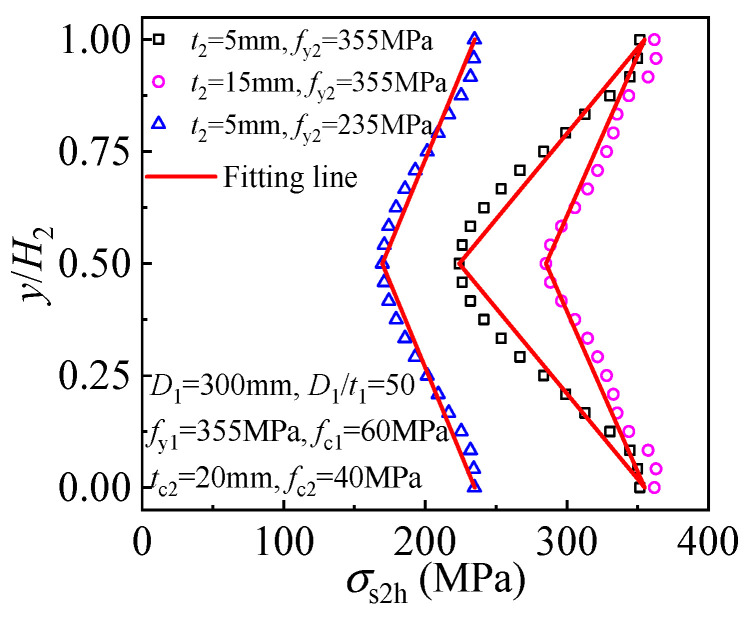
Outer tube hoop stress distribution.

When the load reaches the compressive strength of the component in T-CFST columns of different thicknesses and yield strengths, the variation of the outer tube hoop stress along the height of the outer layer is shown in Figure 10, in which *y* represents the distance of the specific cross-section from the bottom surface of the external jacketing. At the top surface of the external jacketing, the outer tube bears no axial load, and its hoop stress also can reach yield strength. As the length of the friction force transmission gradually increases, the longitudinal stress of the outer tube continuously increases while the hoop stress decreases correspondingly. Also, a linearly decreased outer tube hoop stress is obtained for the T-CFST columns.

## 5. Mechanical Properties of the Cross-Sectional FE Model

The cross-sectional FE model is beneficial to obtain a deep insight into the column’s compressive behavior and develop the methods to predict the compressive strength.

### 5.1. Configuration

Three parts, including the internal CFST, sandwich layer, and outer tube, comprise a typical T-CFST column. Among these, the CFST directly bears the applied load while the outer tube offers lateral confinement. The sandwich material is filled between the internal CFST and outer tube, which is designed to coordinate the radial deformation of different portions and transfer confinement. A systematic analysis based on the established and verified cross-sectional FE model was conducted to determine the suitable sandwich material, and suitable design recommendations for the sandwich material were proposed.

#### 5.1.1. Sandwich Layer Thickness

**Figure 11:**
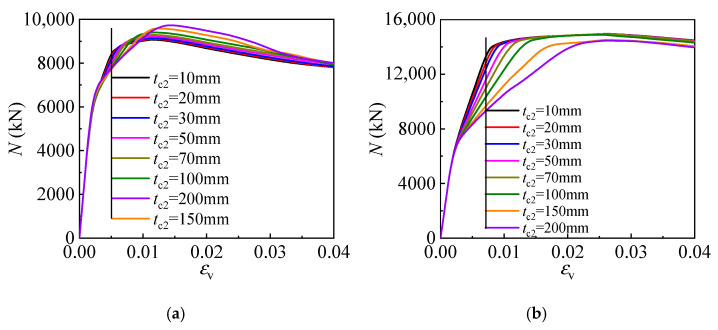
Effect of sandwich layer thickness: (**a**) the outer tube thickness is 5 mm; (**b**) the outer tube thickness is 15 mm.

Keeping the outer tube ratio the same and increasing the diameter results in decreasing thickness, leading to weaker concrete confinement. Therefore, to clarify the influence of the sandwich layer thickness, the outer tube thickness is kept consistent in this section. The influence of the sandwich layer thickness on the columns with different outer tube thicknesses *t*_2_ (5 mm and 10 mm) is shown in Figure 11, where *t*_c2_ is the sandwich layer thickness. At the very beginning of loading, the columns employing sandwich layers of different thicknesses behave similarly. However, the column achieves a lower stiffness at the plastic stage as the sandwich layer thickness increases. After the peak load, the columns with outer tubes of different thicknesses behave in different ways as the sandwich layer thickness changes. Specifically, for the column with a thinner outer tube (*t*_2_ = 5 mm), increasing the sandwich layer thickness leads to improved strength but has a marginal effect on the stiffness.

**Figure 12:**
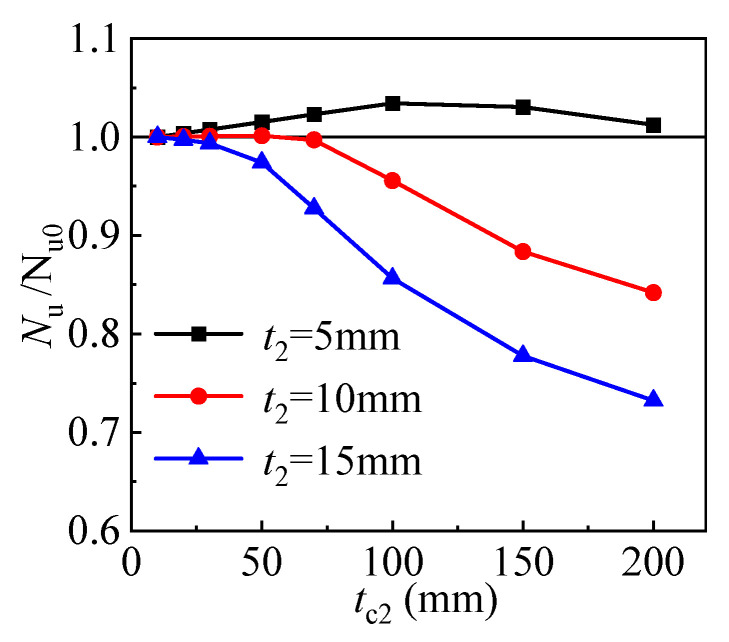
Effect of the sandwich layer thickness.

The compressive strength is given and compared in Figure 12 for columns with different thicknesses of sandwich layer and outer tube. *N*_u0_ is the compressive strength of the column with a sandwich layer thickness of 10 mm. Within a certain range of sandwich layer thickness, the strength of the columns does not correlate with this thickness. However, when the sandwich layer thickness exceeds this range, the further increase in thickness will significantly reduce the bearing capacity. For the T-CFST columns employing outer tubes of different thicknesses, there is also a difference in this range. Specifically, for columns with outer tube thicknesses of 5 mm, 10 mm, and 15 mm, the range limit values of this sandwich layer are 100 mm, 70 mm, and 30 mm, respectively. Due to the sandwich layer in the T-CFST column mainly transferring the confinement, the sandwich layer thickness is suggested to be reduced as much as possible while ensuring pouring quality. Within the scope in this paper, a thickness of no more than 30 mm is suggested.

#### 5.1.2. Sandwich Material Strength

**Figure 13:**
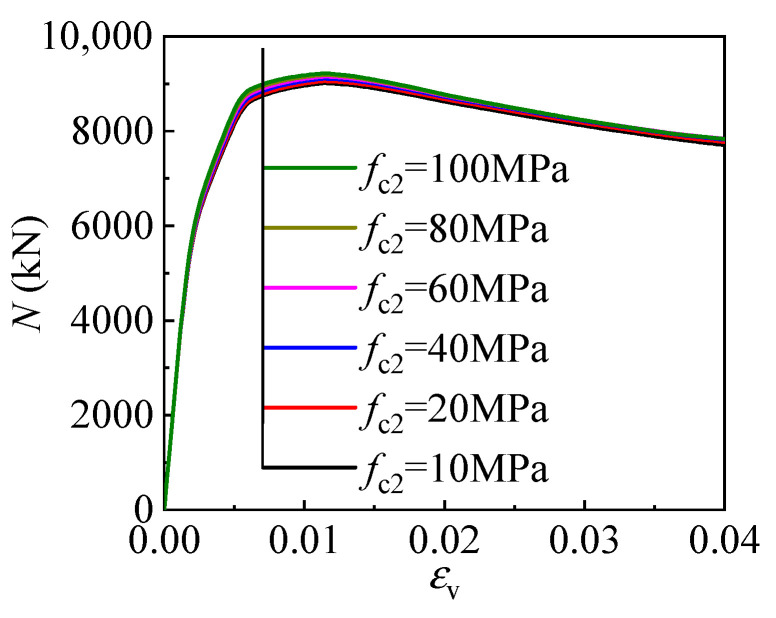
Effect of the sandwich material strength.

The sandwich material fills the gap between the internal CFST and outer tube, coordinating their radial deformation and bearing their radial compression. To clarify the influence of the sandwich material strength for the columns employing different strength sandwich materials is compared in Figure 13. The sandwich material strength has a margin effect on the column’s compressive behavior. Specifically, increasing the sandwich material strength leads to a slightly reduced compressive strength of the T-CFST column, but the decrease is within 2.5%, indicating low-strength materials can be utilized as sandwich material to further reduce the column’s cost.

### 5.2. Parameter Study

To quantify the key parameters of the T-CFST column on its cross-sectional compressive strength, a systematic parameter study is conducted based on the developed and verified cross-sectional FE model. The considered parameter includes the outer tube thickness *t*_2_, inner tube diameter-to-thickness ratio *D*_1_/*t*_1_, concrete strength *f*_c1_, yield strength of outer tube *f*_y2_ and inner tube *f*_y1_, and the detail values of different parameters are listed in Table 2. The diameter of the inner and outer tube is 300 mm and 350 mm, respectively, and the sandwich material axial compressive strength is 40 MPa.

#### 5.2.1. Outer Tube Thickness

**Figure 14:**
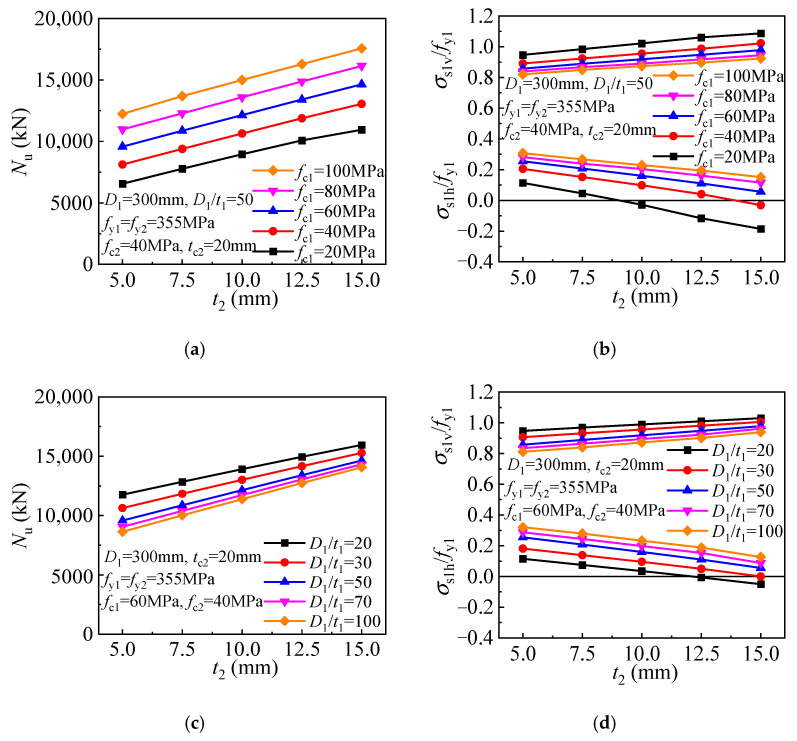
Influence of outer tube thickness: (**a**) Compressive strength (*D*_1_/*t*_1_ = 50); (**b**) Inner tube stress (*D*_1_/*t*_1_ = 50); (**c**) Compressive strength (*f*_c1_ = 60 MPa); (**d**) Inner tube stress (*f*_c1_ = 60 MPa).

The enhanced strength of the T-CFST column is derived from the confinement by the outer tube. Figure 14 depicts the strength and corresponding inner tube stress for the FE models employing outer tubes of different thicknesses. The outer tube does not develop the longitudinal stress, and a hoop stress of yield strength is obtained at the compressive strength. The confinement can be enhanced greatly by increasing the outer tube thickness. Hence, keeping the inner tube diameter-to-thickness ratio unchanged, the strength is linearly improved with the increase of the outer tube thickness for the T-CFST columns with different core concrete strengths employed (Figure 14a). In addition, the inner tube hoop stress decreases while the longitudinal stress increases (Figure 14b). The inner tube may be remains compressed horizontally at the compressive strength for the columns employing lower strength concrete (*f*_c1_ ≤ 40 MPa), and the longitudinal stress may exceed the yield strength. For the T-CFST columns with inner tubes of different diameter-to-thickness ratios, the strength is also linearly improved as the outer tube thickness increases (Figure 14c). Meanwhile, as the outer tube thickness increases, the inner tube hoop stress gradually decreases linearly while its longitudinal stress increases linearly. When a thicker outer tube is employed, the inner tube is also compressed horizontally at the column’s compressive strength, and its longitudinal stress will also exceed the inner tube yield strength (Figure 14d).

#### 5.2.2. Inner Tube Diameter-to-Thickness Ratio

**Figure 15:**
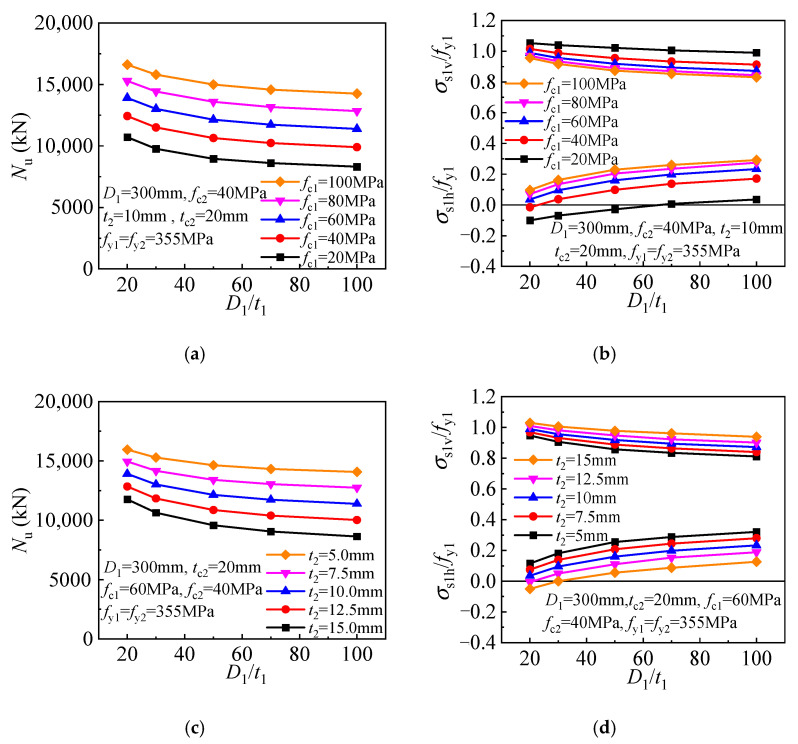
Influence of the inner tube diameter-to-thickness ratio: (**a**) Compressive strength (*t*_2_ = 10 mm); (**b**) Inner tube stress (*t*_2_ = 10 mm); (**c**) Compressive strength (*f*_c1_ = 60 MPa); (**d**) Inner tube stress (*f*_c1_ = 60 MPa).

A significantly enhanced core concrete is achieved for the T-CFST column owing to the dual lateral confinement by the inner and outer tubes. The compressive strength and the inner tube stress components are given in Figure 15 to determine the effect of inner tube diameter-to-thickness ratio. The steel ratio of the inner tube decreases as the ratio increases, and the direct axial load bearing and concrete confinement also decrease, resulting in an inverse proportionally decreasing compressive strength (Figure 15a). Meanwhile, the inner tube hoop stress gradually while the longitudinal stress decreases as the ratio increases (Figure 15b). The relationship between the compressive strength of T-CFST columns utilizing outer tubes of different inner tube steel ratios is compared in Figure 15c. With an increase in the inner tube diameter-to-thickness ratio, the compressive strength also basically decreases in an inverse proportional pattern, and the inner tube hoop stress also increases while its longitudinal stress gradually decreases. The above analysis reveals that although increasing the inner tube diameter-to-thickness ratio will increase its hoop stress, the concrete confinement by the inner tube is reduced. Furthermore, the increase in inner tube hoop stress will also reduce its longitudinal stress and load-bearing capacity. As a result, the T-CFST column’s compressive strength is continuously decreased owing to the weakened concrete confinement and load-bearing capacity of the inner tube.

#### 5.2.3. Concrete Core Strength

**Figure 16:**
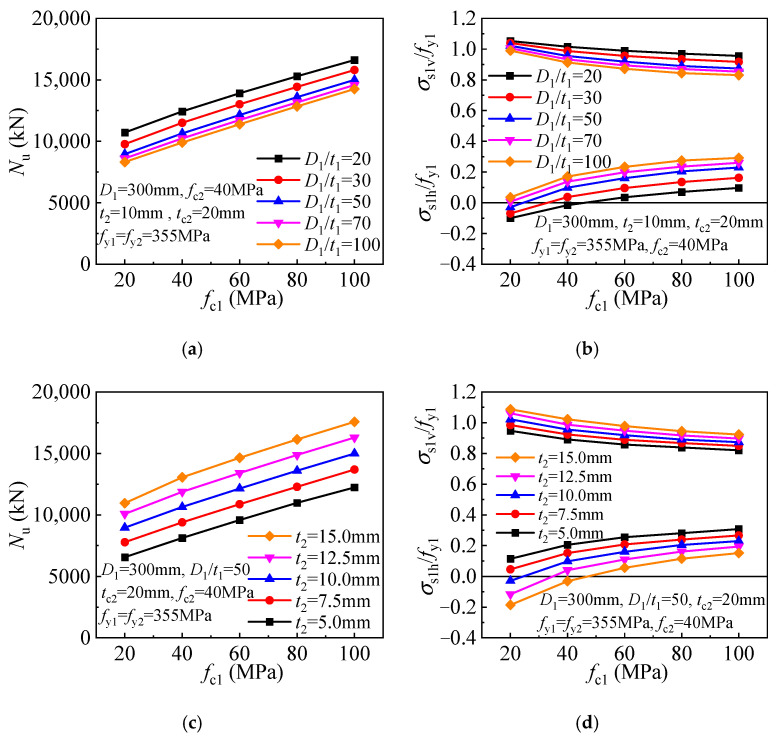
Influence of concrete strength: (**a**) Compressive strength (*t*_2_ = 10 mm); (**b**) Inner tube stress (*t*_2_ = 10 mm); (**c**) Compressive strength (*D*_1_/*t*_1_ = 50); (**d**) Inner tube stress (*D*_1_/*t*_1_ = 50).

An improved compressive strength is obtained for ordinary CFST columns employing concrete of higher strength. Similarly, a linear improvement in compressive strength is obtained for the T-CFST columns employing a higher-strength concrete core (Figure 16a). Meanwhile, a more brittle behavior is expected for the higher-strength concrete, and the inner tube hoop stress is thus increased, leading to enhanced concrete confinement. However, the inner tube longitudinal stress is decreased gradually, leading to a slight reduction in its load-bearing capacity (Figure 16b). As the concrete core strength increases, the compressive strength of the T-CFST column is increased linearly (Figure 16c). Furthermore, the inner tube hoop stress is increased with the increase of the concrete strength, while its longitudinal stress is decreased (Figure 16d).

#### 5.2.4. Outer Tube Yield Strength

**Figure 17:**
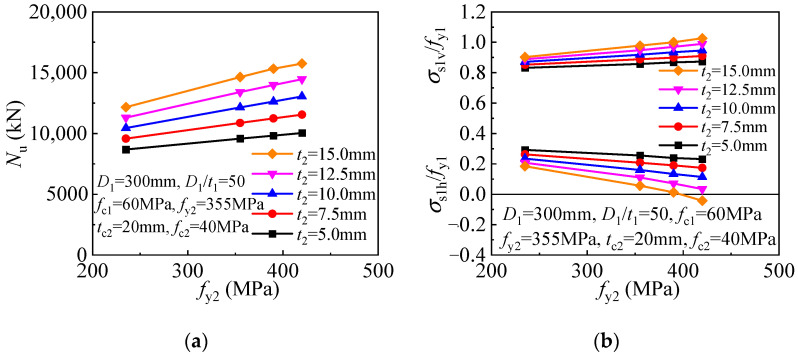
Influence of outer tube yield strength: (**a**) Compressive strength; (**b**) Inner tube stress.

Besides the outer tube thickness, its yield strength is also critical in determining concrete confinement. As the outer tube yield strength increases, more effective confinement is offered to the internal CFST, and the compressive strength of the T-CFST columns is thus increased linearly (Figure 17a). Meanwhile, the decreased inner tube hoop stress is obtained for the columns employing thicker outer tube, and even compressed stress can be developed for the inner tube, leading to improved inner tube longitudinal stress and compressive strength of the members (Figure 17b).

#### 5.2.5. Inner Tube Yield Strength

**Figure 18:**
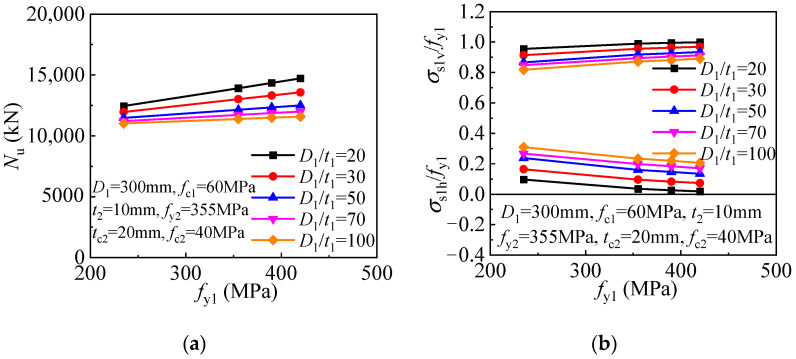
Influence of inner tube yield strength on the cross-sectional mechanical properties: (**a**) Compressive strength; (**b**) Inner tube stress.

The compressive strength and inner tube stress components are given in Figure 18 for the T-CFST columns. The T-CFST column’s compressive strength can be improved linearly as the inner tube yield strength increases (Figure 18a). Meanwhile, increasing the yield strength of the inner tube leads to an enhanced inner tube longitudinal stress but reduced inner tube hoop stress (Figure 18b).

### 5.3. Cross-Sectional Compressive Strength

Based on the parameter study on the cross-sectional FE model under axial compression, the calculation methods for the hoop stress of the inner and outer tubes, and the inner tube longitudinal stress are proposed. The concrete strength under dual confinement thus can be determined. After that, the compressive strength is obtained for the T-CFST cross-section.

**Figure 19:**
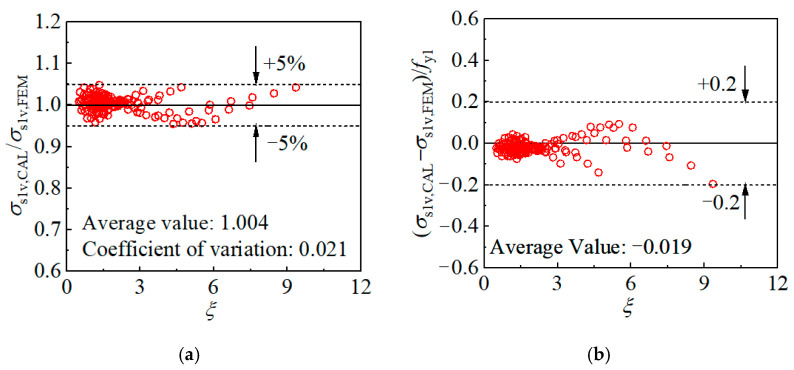
Validation of the inner tube stress calculation method: (**a**) Longitudinal stress; (**b**) Hoop stress.

The outer tube hoop stress is taken as the yield strength *f*_y2_. Based on the stress components of the inner tube obtained by the cross-sectional FE model, the calculation methods are proposed for the inner tube longitudinal and hoop stress, as shown in Equations (3) and (4), respectively. The inner tube stress components with different parameters are thus determined and compared with the results of the FE model (Figure 19), where *σ*_s1v, CAL_, and *σ*_s1h, CAL_ represent the inner tube longitudinal and hoop stress calculated by the formulas, while *σ*_s1v, FEM_, and *σ*_s1h, FEM_ are corresponding value determined by the cross-sectional FE model. The proposed calculation methods are satisfactory in predicting the inner tube stress components. Specifically, the difference between the inner tube longitudinal stress determined by the equation and the FE model is within 5%, with an average value of 1.004 and a coefficient of variation of 0.021. In addition, the difference between the inner tube hoop stress predicted by the equation and the FE model is less than 0.2*f*_y1_, with an average value of 0.019*f*_y1_.

(3) $$\sigma_{s1v}=(0.0018t_{2}f_{y2}+45.8328)(0.0003f_{y1}/(D_{1}/t_{1})+0.0359)(-0.0008f_{c1}+0.5143)f_{y1}$$

(4) $$\sigma_{s1h}=\left(\sqrt{4f_{y1}^{2}-3\sigma_{s1v}^{2}}-\sigma_{s1v}\right)/2$$

**Figure 20:**
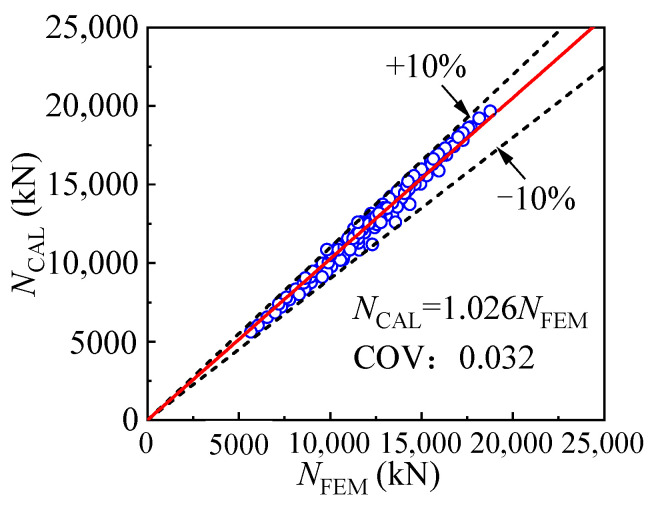
Comparison of cross-sectional compressive strength obtained by equation and FE model.

Based on the calculation methods for the outer and inner tube stress components, the prediction method is proposed for the cross-sectional compressive strength, as shown in Equation (5). *f*_cc1_ represents the confined concrete compressive strength, which is determined by Equation (6); *p* is the concrete confining stress, and it can be calculated by Equation (7). The T-CFST column’s cross-sectional strength is determined and given in Figure 20, where *N*_CAL_ and *N*_FEM_ represent the compressive strength obtained by the calculation method and FE model, respectively. The difference is within 10%, with a mean ratio being 2.6% and a coefficient of variation being 0.032, indicating that the proposed calculation method is precise enough.

(5) $$N=f_{\mathrm{cc}1}A_{\;c1}+\sigma_{s1v}A_{\;s1}$$

(6) $$f_{\mathrm{cc}1}=\left(1+3.5{\left(\frac{p}{f_{c1}}\right)}^{0.85}\right)f_{c1}$$

(7) $$p=\left(2f_{y2}t_{2}+2\sigma_{s1h}t_{1}\right)/(D_{1}-2t_{1})$$

## 6. Mechanical Properties of FE Model for Stub Column

The influence of the interfacial friction is discussed in the following and the mechanical properties of the T-CFST stub columns are further determined.

### 6.1. Configuration

To make it easier to connect the beam and CFST column, an internal or external steel plate is welded to the CFST column in the beam-column joint area, which has an effective constraint on the CFST column end. The influence of the reduction of the external jacketing at the column ends and the strong local constraint by the connecting steel plate at the T-CFST column end is quantitatively studied based on the developed FE model for the stub column, then a suitable column end’s configuration is suggested.

**Figure 21:**
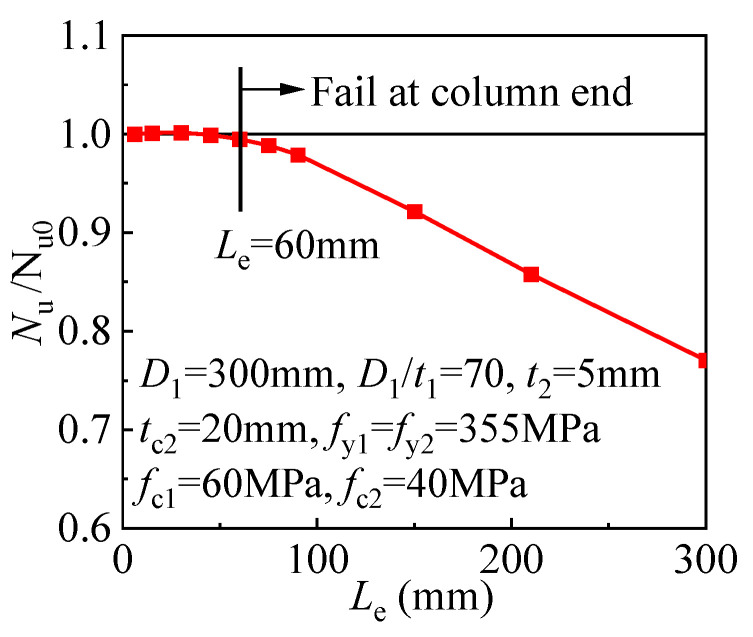
Influence of end length.

The effect of the column end length is shown in Figure 21, where *N*_u0_ is the compressive strength for the T-CFST columns when the end length is 5 mm. The connecting steel plate at the column effectively strengthens the column end area when the end length is within 60 mm. As a result, the column fails at the column mid-region, and the compressive strength almost remains unchanged. However, if the column end length exceeds 60 mm, the connecting steel plate is not enough to constrain the column end area, and the column thus fails at the column end, resulting in a rapid reduction of the compressive strength. The above analysis indicates that decreasing the end length of the T-CFST stub column is beneficial to fully develop its compressive behavior, but the column end length should not be too small to allow the longitudinal relative slip between the internal CFST and external jacketing. Therefore, the T-CFST stub column end length is suggested to be less than 60 mm within the scope of this paper. For the T-CFST column with an end length exceeding 60 mm, additional strengthening measures should be employed to fully develop its compressive strength, and the experimental study has shown that CFRP wrapping at the column end is efficient and convenient [37].

### 6.2. Parameter Study

Based on the developed and validated FE model of the stub column, the influence of key parameters is quantitatively analyzed. The FE model’s dimensions and parameters are the same as those in the cross-sectional FE model, and detailed information is given in Section 5.2. The height-to-diameter ratio of the stub column is taken as three, and the column end length is adopted as 50 mm.

#### 6.2.1. Outer Tube Thickness

**Figure 22:**
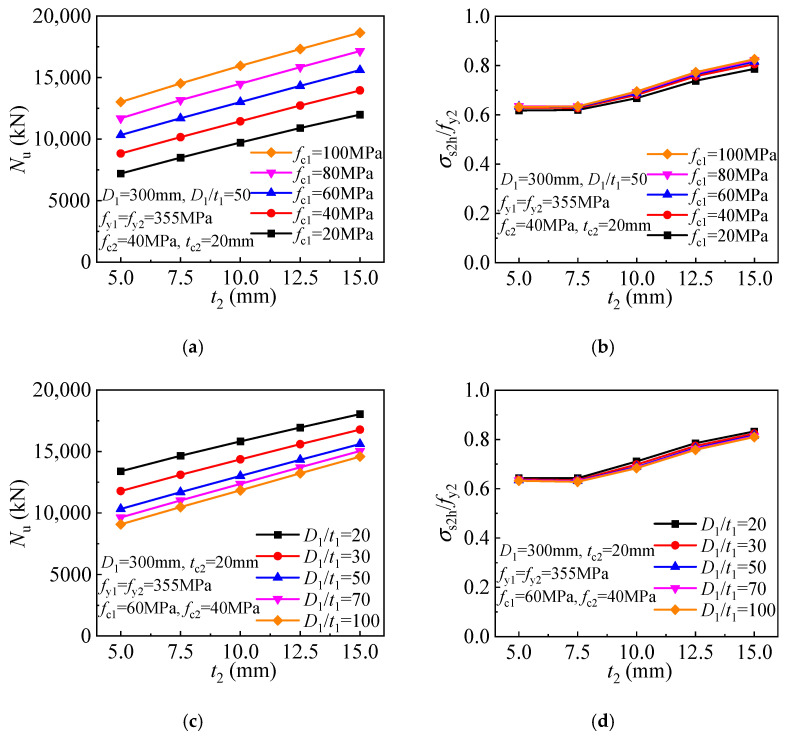
Mechanical properties of stub columns with different outer tub thicknesses: (**a**) compressive strength (*D*_1_/*t*_1_ = 50); (**b**) outer tube hoop stress (*D*_1_/*t*_1_ = 50); (**c**) compressive strength (*f*_c1_ = 60 MPa); (**d**) outer tube hoop stress (*f*_c1_ = 60 MPa).

Increasing the outer tube thickness is effective in improving concrete confinement, leading to a significantly enhanced compressive strength (Figure 22). As the outer tube thickness increases, the compressive strength is increased linearly, as shown in Figure 22a. The mid-height outer tube hoop stress corresponding to the compressive strength is given in Figure 22b. For the stub column with an outer tube thickness of no more than 7.5 mm, the outer tube hoop stress remains unchanged. However, the outer tube hoop stress increases linearly as its thickness increases when the thickness exceeds 7.5 mm. A linearly increased compressive strength is obtained as the outer tube thickness increases (Figure 22c). Furthermore, the outer tube hoop stress is also increased linearly as the outer tube thickness increases, when its thickness is larger than 7.5 mm, as depicted in Figure 22d.

#### 6.2.2. Inner Tube Diameter-to-Thickness Ratio

**Figure 23:**
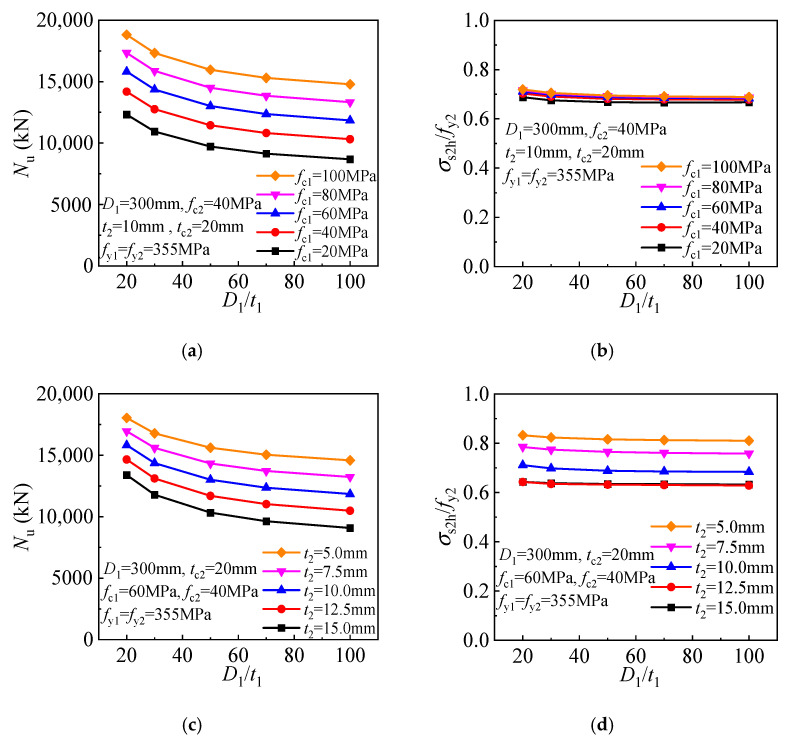
Influence of the inner tube diameter-to-thickness ratio: (**a**) compressive strength (*t*_2_ = 10 mm); (**b**) outer tube hoop stress (*t*_2_ = 10 mm); (**c**) compressive strength (*f*_c1_ = 60 MPa); (**d**) outer tube hoop stress (*f*_c1_ = 60 MPa).

The steel ratio of the inner tube decreases as the diameter-to-thickness ratio increases, and the direct axial load bearing and concrete confinement also decrease, resulting in an inverse proportionally decreasing compressive strength (Figure 23a). In addition, the increasing inner tube diameter-to-thickness ratio also leads to a slightly decreased outer tube hoop stress (Figure 23b). The relationship between the stub column’s compressive strength and the inner tube diameter-to-thickness ratio is showed as follows. As the inner tube diameter-to-thickness ratio increases, the compressive strength also decreases in an inversely proportional manner, as depicted in Figure 23c. At the same time, the mid-height outer tube hoop stress decreases slightly as the inner tube diameter-to-thickness ratio increases (Figure 23d).

#### 6.2.3. Concrete Strength

**Figure 24:**
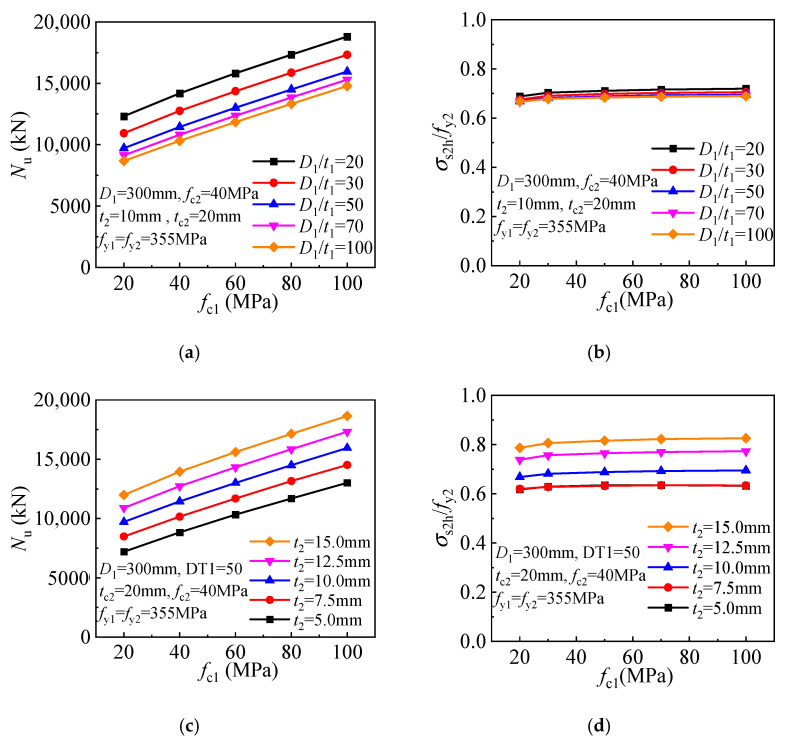
Mechanical properties of stub columns with core concrete of different strengths: (**a**) compressive strength (*t*_2_ = 10 mm); (**b**) outer tube hoop stress (*t*_2_ = 10 mm); (**c**) compressive strength (*D*_1_/*t*_1_ = 50); (**d**) outer tube hoop stress (*D*_1_/*t*_1_ = 50).

To clarify the effect of the concrete strength, Figure 24 depicts the relationship between the compressive strength of the stub column and the concrete strength. Improved concrete strength leads to a linearly increased compressive strength for the T-CFST stub columns (Figure 24a) and a slightly increased outer tube hoop stress (Figure 24b). For the T-CFST columns employing outer tubes of different thicknesses, the compressive strength also increases linearly, while the outer tube hoop stress increases slightly, as shown in Figure 24c,d, respectively.

#### 6.2.4. Outer Tube Yield Strength

**Figure 25:**
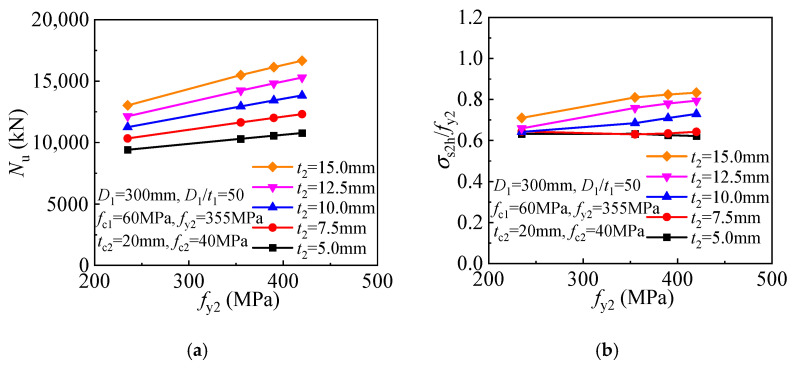
Mechanical properties of stub columns with outer tubes of different yield strengths: (**a**) compressive strength; (**b**) outer tube hoop stress.

The effect of the outer tube yield strength gradually enhances as its thickness increases (Figure 25a). When the outer tube thickness is within 7.5 mm, its yield strength almost has no influence on the ratio of the outer tube hoop stress and the yield stress *σ*_s2h_/*f*_y2_. However, when the thickness exceeds 7.5 mm, an improved ratio of the outer tube hoop stress and yield strength *σ*_s2h_/*f*_y2_ is achieved as the outer tube yield strength increases.

#### 6.2.5. Inner Tube Yield Strength

**Figure 26:**
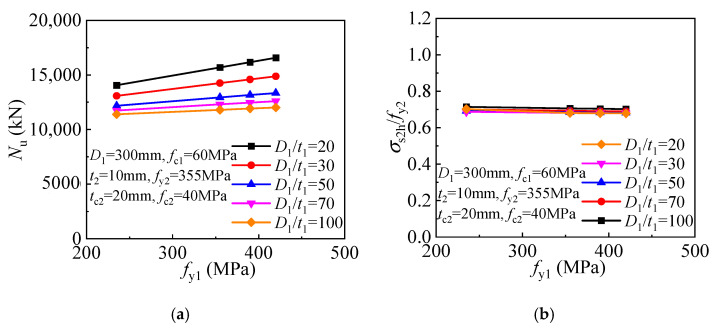
Effect of inner tube yield strength: (**a**) compressive strength; (**b**) outer tube hoop stress.

As the inner tube yield strength increases, a linearly enhanced compressive strength is obtained for the T-CFST stub column. In addition, the magnitude of the improvement in compressive strength gradually increases as the inner tube diameter-to-thickness ratio decreases. (Figure 26a). Meanwhile, as the inner tube yield strength increases, the outer tube hoop stress slightly decreases (Figure 26b).

### 6.3. T-CFST Stub Column Compressive Strength

The effect of the interfacial friction on the stress development of each portion is quantitively studied based on the parameter study, and suitable calculation methods are proposed correspondingly. Finally, the prediction method is derived for the compressive strength of the stub column.

#### 6.3.1. External Jacketing Bearing Capacity

The axial compressive loading test shows that the external jacketing will slide relative to the internal CFST before reaching the column compressive strength [13,37]. Therefore, the interfacial friction is assumed to follow the Coulomb friction principle, in which the friction stress is proportional to the normal compressive stress. The sandwich layer develops evenly distributed radial cracks before the column’s compressive strength, and its confinement is thus ignored. Therefore, the interfacial normal compressive stress *p*_s2s1_(*y*) can be determined by the outer tube hoop stress *σ*_s2h_(*y*) only, as shown in Equation (8), where *y* is the distance from the specific cross-section to the external jacketing.

(8) $$p_{s2s1}(y)=2\sigma_{s2h}(y)t_{2}/D_{1}$$

Due to the friction over the external jacketing and the internal CFST interface, the outer tube hoop stress is not uniform. The analysis in Section 4.2 shows that the outer tube hoop stress reaches the steel material yield strength at the external jacketing end cross-section and then decreases linearly to the column mid-height. With the determination of the outer tube hoop stress at the column mid-height *σ*_s2h,m_ following Equation (9), the variation of the stress along the external jacketing height can thus be determined.

(9) $$\sigma_{s2h,m}=(0.0300t_{2}+3.1490)(0.00001t_{2}f_{y2}+0.1650)f_{y2}$$

**Figure 27:**
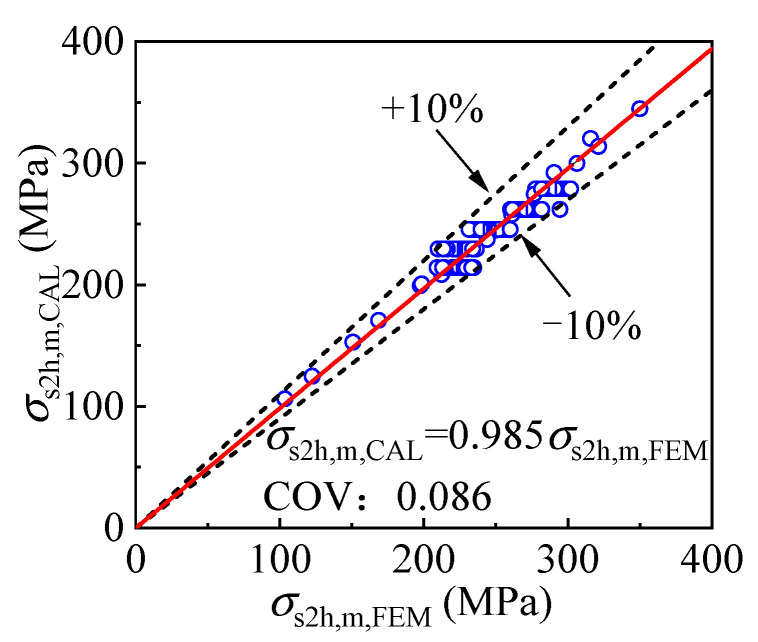
Validation of the mid-height cross-sectional outer tube hoop stress prediction method.

The outer tube hoop stress obtained by the equation *σ*_s2h,m, CAL_ and the FE model *σ*_s2h,m, FEM_ is given and compared in Figure 27. The difference between *σ*_s2h,m, CAL_ and *σ*_s2h,m, FEM_ is within 10%, and the average of *σ*_s2h,m, CAL,_ and *σ*_s2h,m, FEM_ is 0.985, with a coefficient of variation of 0.086, indicating the equation is precise enough.

Based on the above analysis, the outer tube hoop stress along the external jacketing height can be determined by Equation (10). The axial load sustained by the external jacketing can be determined by Equation (11). Meanwhile, the axial load sustained by the external jacketing should not exceed its load-bearing capacity, which can be calculated by Equation (12), in which *f*_cc2_ is the confined sandwich material strength and can be determined following Equation (13). *p*_s2c2_ is the sandwich material confining stress at the external jacketing mid-height cross-section, which can be calculated by Equation (14). With the above parameters determined, the load sustained by the external jacketing *N*_f_ thus can be adopted by Equation (15).

(10) $$\sigma_{s2h}(y)=f_{y2}-2y(f_{y2}-\sigma_{s2h,m})/H$$

(11) $$N_{\;f1}=\pi \mu D_{1}\int_{0}^{H/2}p_{s2s1}(y)dy=\frac{1}{2}\pi \mu t_{2}H(f_{y2}+\sigma_{s2h,m})$$

(12) $$N_{f2}=f_{\mathrm{cc}2}A_{c2}+f_{y2}A_{s2}$$

(13) $$f_{\mathrm{cc}2}=\left(1+3.5{\left(\frac{p_{s2c2}}{f_{c2}}\right)}^{0.85}\right)f_{c2}$$

(14) $$p_{s2c2}=2\sigma_{s2h,m}t_{2}/(D_{2}-2t_{2})$$

(15) $$N_{f}=\min(N_{f1},N_{f2})$$

#### 6.3.2. Internal CFST Bearing Capacity

**Figure 28:**
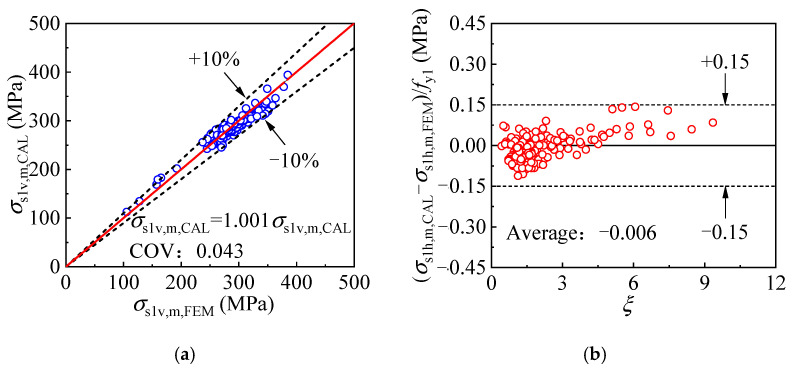
Comparison of the mid-height cross-sectional inner tube stress obtained by the equation and the FE model: (**a**) longitudinal stress; (**b**) hoop stress.

The axial load sustained by the internal CFST portion can be determined by considering the contribution of both the inner tube and core concrete, and the inner tube longitudinal stress *σ*_s1v,m,_ and hoop stress *σ*_s1h,m_ can be calculated by Equations (16) and (17), respectively. The comparison of the inner tube stress components obtained by the equations and the FE model is depicted in Figure 28, where *σ*_s1v,m, CAL_ and *σ*_s1v,m, FEM_ is the longitudinal stress determined by the formula and FE model, while *σ*_s1h,m, CAL_ and *σ*_s1h,m, FEM_ is the hoop stress given by the corresponding methods. The difference between *σ*_s1v,m, FEM_ and *σ*_s1v,m, CAL_ is no more than 10%, with the average ratio of the two values being 1.001 and the coefficient of variation being 0.043. Meanwhile, the difference between the inner tube hoop stress determined by the equation and the FE model is less than 0.15*f*_y2_, with an average error of 0.00 *f*_y2_. The above comparison reveals that the calculation methods are satisfactory in calculating the stress components of the mid-height inner tube.

(16) $$\begin{array}{cc} \sigma_{\mathrm{slv},m}= & 0.89\sigma_{\mathrm{slv}}\\ = & 0.89\;\times \;(0.0018t_{2}\sigma_{s2h,m,\mathrm{CAL}}\;+\;45.8328)(0.0003f_{y1}/(D_{1}/t_{1})+0.0359)\\ \; & (-0.0008f_{c1}\;+\;0.5143)f_{y1} \end{array}$$

(17) $$\sigma_{s1h,m}=\left(\sqrt{4f_{y1}^{2}-3\sigma_{s1v,m}^{2}}-\sigma_{s1v,m}\right)/2$$

The concrete confining stress by the inner and outer tubes *p*_c1,m_ can be calculated by Equation (18), and the confined concrete strength can thus be determined by Equation (6), in which *p* needs to be replaced by *p*_c1,m_. Finally, the load sustained by the internal CFST is given by Equation (5), and the inner tube longitudinal stress *σ*_s1v_ is adopted as *σ*_s1v,m_.

(18) $$p_{c1,m}=\left(2\sigma_{s2h,m}t_{2}+2\sigma_{s1h,m}t_{1}\right)/(D_{1}-2t_{1})$$

#### 6.3.3. Compressive Strength of the T-CFST Stub Column

Based on the above calculation methods of the load sustained by the external jacketing, inner tube, and the concrete core, the compressive strength of the T-CFST stub column is given by Equation (19) based on the superposition method.

(19) $$N_{u}=N_{f}+\sigma_{s1v,m}A_{s1}+f_{\;\mathrm{cc}1}A_{c1}$$

**Figure 29:**
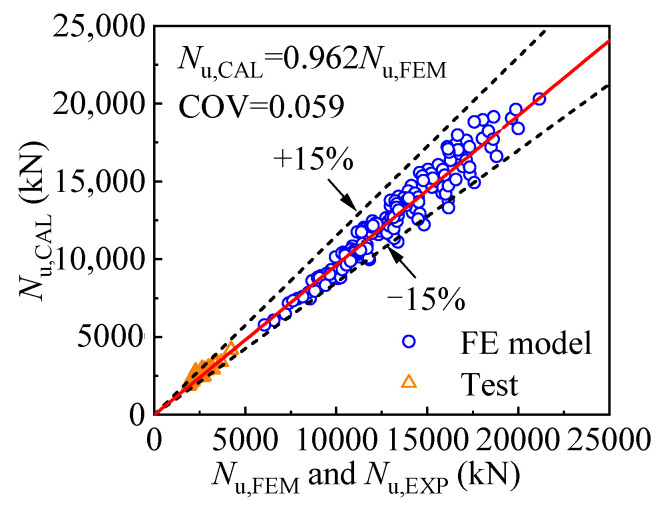
Comparison of the column strength obtained by equation and FE model or test [37].

Figure 29 shows the comparison of the compressive strength obtained by the equation *N*_u, CAL_ and numerical analysis *N*_u, FEM_ as well as experiment *N*_u, EXP_ in reference [37]. The comparison reveals that the average ratio of the calculated result and the results of the FE model is 0.962, and the difference between the two values is less than 15%, with the coefficient of variation being 0.059, indicating the calculation method is accurate enough to predict the compressive strength of the T-CFST stub column. It should be noted that the conclusion is drawn based on the results of the FE models within the scope of this paper.

## 7. Conclusions

In this paper, the FE model is firstly developed for the T-CFST cross-section and stub column; then, the confining mechanism and effect of key parameters on the mechanical properties are clarified; finally, the calculation method is proposed to determine the compressive strength. The following conclusion can be drawn:

(1) The development of concrete confinement by the inner and outer tubes in the T-CFST columns can be divided into the following stages: the internal CFST portion directly sustains the applied axial load while the outer tube does not; once the axial load is applied, the outer tube begins to offer lateral confinement to the internal CFST while the inner tube is compressed horizontally; the horizontal expansion of the core concrete develops quickly with the increase in axial load, leading to an increasing confinement by the outer tube, and the inner tube is still under compression laterally; after the outer tube yields, the confinement by the outer tube stays unchanged, and the inner tube hoop stress gradually turns from compression to tension, leading to continuously increasing concrete confinement.
(2) The lateral confinement by the outer tube is determined by the outer tube steel ratio and yield strength. The variation of the confining pressure by the outer tube along the column height is illustrated through the consideration of the interfacial friction over the interface between the external jacketing and the internal CFST portion. The prediction method considering the confinement by the outer tube is proposed to determine the inner tube hoop stress, and the inner tube longitudinal stress can thus be given based on Mises yielding criterion. The strength and deformation ability of the core concrete is enhanced significantly by the effective confinement of the outer and inner tubes, and a calculating equation is also proposed to determine the strength of the core concrete at the compressive strength of the T-CFST columns.
(3) Considering the distribution of the interfacial friction and its transferring length, the axial load carried by the external jacketing can be determined; based on the composite action between the external jacketing, inner tube, and the core concrete, a suitable calculation method is proposed to determine the compressive strength of the T-CFST columns.

## Figures and Tables

**Table 1:** Parameters of the cross-sectional FE models.

FE Model	*D*_1_ (mm)	*D*_1_/*t*_1_	*D*_2_ (mm)	*D*_2_/*t*_2_	*f*_y1_ (MPa)	*f*_y2_ (MPa)	*f*_c1_ (MPa)	*f*_c2_ (MPa)	*α*_1_ (%)	*α*_2_ (%)	*α* (%)	*ξ*
T-CFST	300	50	350	100	355	355	60	40	8.5	5.8	14.4	0.85
CFST-1	300	50	—	—	355	355	60	40	8.5	—	8.5	0.50
CFST-2	300	30	—	—	355	355	60	40	14.8	—	14.8	0.88

**Table 2:** Parameters value.

Parameter	Value
Outer tube thickness *t*_2_ (mm)	5, 7.5, 10, 12.5, 15
Inner tube diameter-to-thickness ratio *D*_1_/*t*_1_	20, 30, 50, 70, 100
Concrete strength *f*_c1_ (MPa)	20, 40, 60, 80, 100
Outer tube yield strength *f*_y2_ (MPa)	235, 355, 420
Inner tube yield strength *f*_y1_ (MPa)	235, 355, 420

## Data Availability

All data included in this study are available upon request by contact with the corresponding author.
