# Correction: Li et al. Numerical Study on the Axial Compressive Behavior of Steel-Tube-Confined Concrete-Filled Steel Tubes. *Materials* 2024, *17*, 155

**DOI:** 10.3390/ma17051132

**Published:** 2024-02-29

**Authors:** Xiaozhong Li, Sumei Zhang, Yu Tao, Bing Zhang

**Affiliations:** 1 School of Civil and Environmental Engineering, Harbin Institute of Technology (Shenzhen), Shenzhen 518055, China; lixiaozhong@stu.hit.edu.cn (X.L.); 19b954014@stu.hit.edu.cn (Y.T.); zhangbing2003@hit.edu.cn (B.Z.); 2 Guangdong Provincial Key Laboratory of Intelligent and Resilient Structures for Civil Engineering (Shenzhen), Shenzhen 518055, China

In the original publication [1], there was a mistake in Figures 17 and 18 as published. Figures 17 and 18 were mistakenly placed in the original publication. The authors have carefully checked the data again and re-drawn the graphs. The graphs illustrate the relationships between *N*u and the parameters; the new graphs are now in agreement with the statement in the corresponding paragraph in the original publication. The corrected Figure 17 and Figure 18 appear below. The authors state that the scientific conclusions are unaffected. This correction was approved by the Academic Editors. The original publication has also been updated.

## Figures and Tables

**Figure 17:**
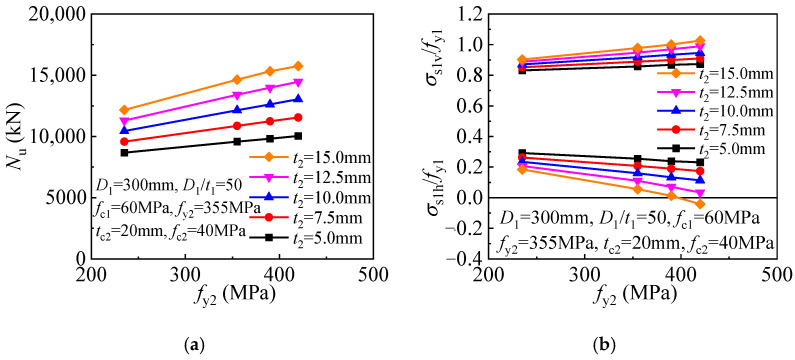
Influence of outer tube yield strength: (**a**) compressive strength; (**b**) inner tube stress.

**Figure 18:**
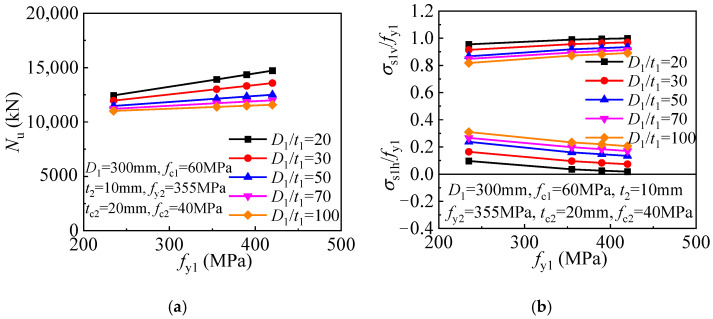
Influence of inner tube yield strength on the cross-sectional mechanical properties: (**a**) compressive strength; (**b**) inner tube stress.
